# Analysis of Demographic Determinants in the Selection of Implant-Supported Prostheses: A Retrospective Study of 984 Patients Treated at a Spanish University Clinic

**DOI:** 10.4317/jced.63714

**Published:** 2026-01-28

**Authors:** Diego Gómez-Costa, Rocío Cascos-Sánchez, José Luis Antonaya-Martín, Noelia Rivas-Martín, Pablo Lastra-Prados

**Affiliations:** 1 Master’s Degree in Implant-Supported Prostheses, Rey Juan Carlos University (URJC); 2 Lecturer in the Master’s Programme in Implant-Supported Prostheses, URJC; 3 Doctor of Dentistry, Director of the Master’s Programme in Implant-Supported Prostheses, URJC

## Abstract

**Background:**

Implant-supported prosthetic rehabilitation is currently considered a predictable and effective treatment for partial and complete edentulism. Nevertheless, treatment selection is not exclusively determined by clinical variables, and patient-related demographic factors may also influence therapeutic decisions, particularly in university clinical settings. Objective: To analyse the annual evolution and distribution of implant-supported prosthetic treatments performed at a Spanish university dental clinic and to evaluate the association between patient age and sex and the type of prosthesis selected.

**Material and Methods:**

A retrospective cross-sectional study was conducted including 984 adult patients rehabilitated with implant-supported prostheses between 2018 and 2022. Prostheses were classified according to Misch's classification into fixed and removable categories. Demographic variables included age and sex. Descriptive statistics, bivariate analyses, and multinomial logistic regression were performed.

**Results:**

A progressive annual increase in the number of implant-supported rehabilitations was observed throughout the study period. Single implant-supported crowns were the most frequent rehabilitation (52.4%), followed by implant-supported fixed partial dentures (32.5%). Mean age increased progressively with prosthetic complexity, reaching the highest values among patients rehabilitated with overdentures. Multivariate analysis identified age as the strongest predictor of prosthesis type. Male sex was independently associated with bar-retained overdentures.

**Conclusions:**

Patient age is the primary demographic determinant influencing implant-supported prosthesis selection in a university clinical setting. Older patients show a clear trend towards more extensive fixed and removable rehabilitations. These findings provide relevant epidemiological information for treatment planning and resource management in academic dental centres.

## Introduction

Tooth loss remains a major public health problem worldwide and is associated with functional impairment, aesthetic concerns, reduced quality of life, and psychosocial limitations (1 - 3). Implant-supported prosthetic rehabilitation has become the gold standard for the replacement of missing teeth, demonstrating high long-term survival rates and favourable patient-reported outcomes (4 - 6). The choice of implant-supported prosthetic design-ranging from single crowns to fixed partial dentures, hybrid prostheses, or removable overdentures-is traditionally guided by clinical and anatomical factors such as the extent of edentulism, bone volume, occlusal demands, and systemic health conditions (7 , 8). However, growing evidence suggests that demographic characteristics, particularly age and sex, may influence both the extent of tooth loss and the type of rehabilitation ultimately provided (9 - 11). Age has consistently been associated with cumulative tooth loss and an increased prevalence of partial and complete edentulism, which in turn may necessitate more extensive prosthetic solutions (12 - 14). Sex-related differences in oral health status, behavioural risk factors, and healthcare-seeking patterns have also been described, although their impact on implant prosthetic selection remains controversial (15 , 16). Despite the increasing number of implant treatments performed in university clinics, epidemiological data describing prosthetic treatment patterns in this setting are scarce. University clinics represent a unique environment where clinical decision-making may be influenced by educational objectives, patient socioeconomic profile, and access to care (17 , 18). Hypothesis: Patient age and sex are associated with the type of implant-supported prosthesis selected. Objective: To evaluate the annual evolution and distribution of implant-supported prosthesis types in a Spanish university dental clinic and to analyse the association between patient age and sex and prosthesis selection.

## Material and Methods

1. Study Design and Population A retrospective observational cross-sectional study was conducted at the Rey Juan Carlos University Dental Clinic. All adult patients (18 years) rehabilitated with implant-supported prostheses between January 2018 and January 2022 were eligible. Patients treated exclusively with conventional (non-implant-supported) prostheses, warranty repetitions, or treatments initiated outside the centre were excluded. The final sample comprised 984 patients. 2. Data Collection and Variables Anonymised data were extracted from the electronic clinical records (Cliniwin® software). Dependent variable: Type of implant-supported prosthesis, classified according to Misch (19): Fixed prostheses (FP) FP1: Single crown or fixed partial denture FP2: Fixed complete prosthesis FP3: Hybrid fixed prosthesis Removable prostheses (RP) RP4: Implant-supported bar-retained overdenture RP5: Implant- and mucosa-supported overdenture Independent variables: Age (continuous and categorised into decades) Sex (male/female) Year of treatment (2018-2022) 3. Statistical Analysis Statistical analysis was performed using Stata® 16.1. Descriptive statistics were calculated for all variables. The Kruskal-Wallis test was used to assess age differences between prosthesis groups, and the Chi-square test was applied for sex associations. A multinomial logistic regression model was constructed using FP1 single crowns as the reference category. Statistical significance was set at p 0.05.

## Results

**Figure 1:**
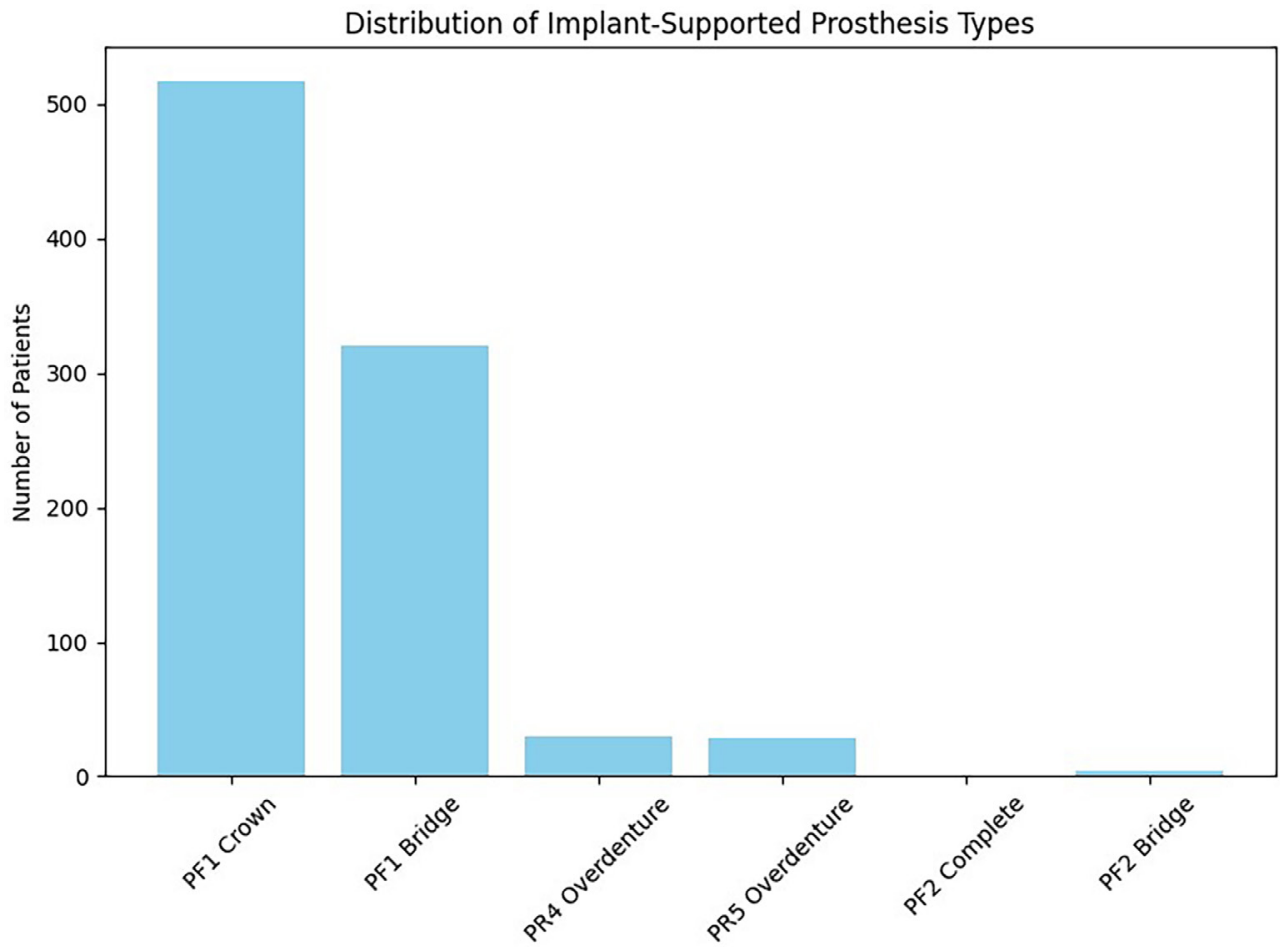
Distribution of Implant-Supported Prosthesis Types.

1. Annual Evolution of Implant-Supported Treatments A steady increase in the number of implant-supported prosthetic treatments was observed over the study period, rising from 150 patients in 2018 to 245 patients in 2022. 2. Distribution of Prosthesis Types Single implant-supported crowns (FP1) were the most frequent rehabilitation (n = 516; 52.4%), followed by implant-supported fixed partial dentures (n = 320; 32.5%). Removable prostheses (RP4 and RP5) accounted for a smaller proportion of treatments. FP2 and FP3 prostheses were infrequent, (Fig. 1).

1

**Figure 2:**
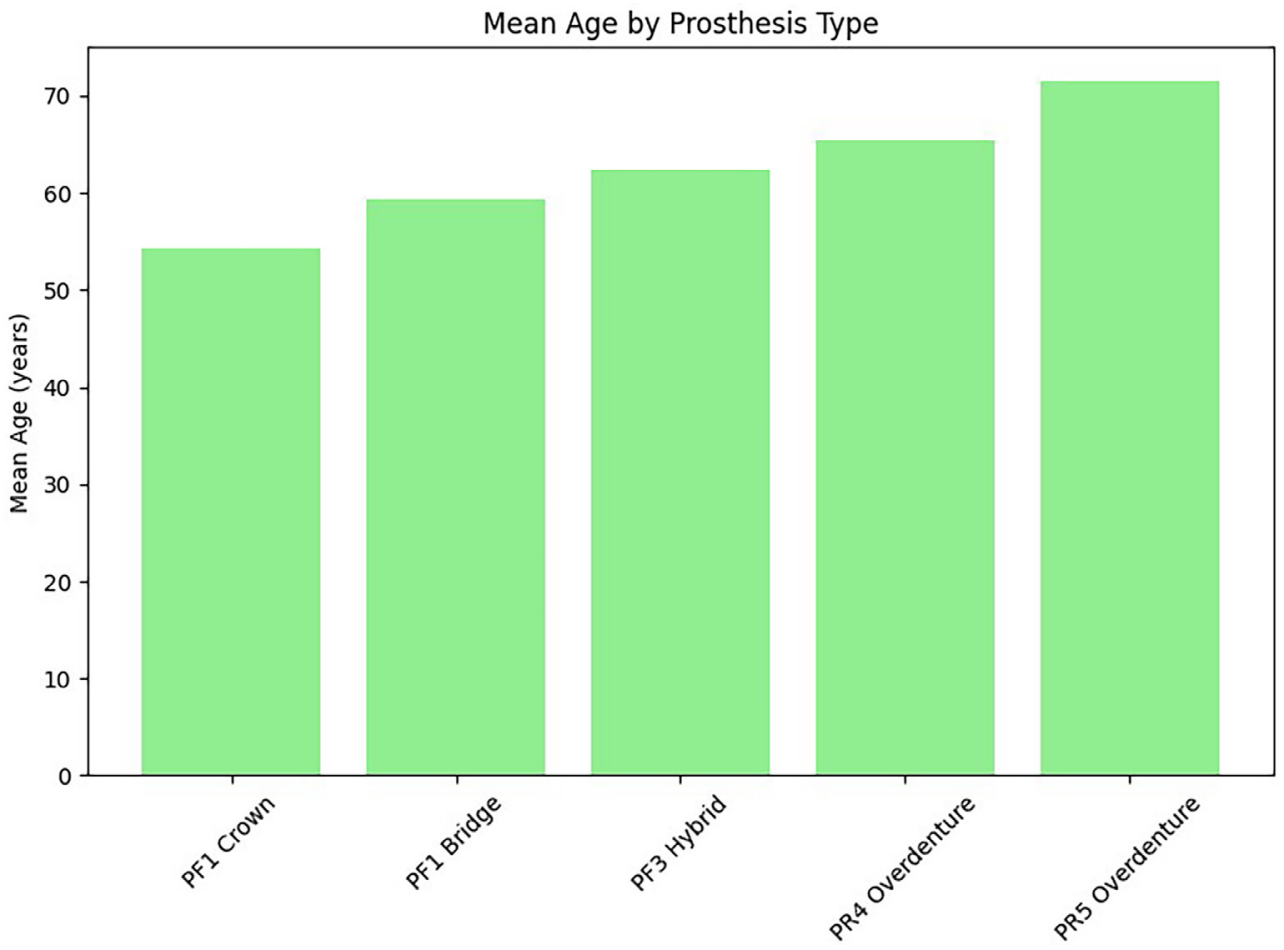
Mean Age by Prosthesis Type.

3. Age and Prosthesis Type Mean patient age increased progressively with prosthetic complexity. Patients rehabilitated with single crowns had the lowest mean age, whereas those treated with overdentures showed the highest values. These differences were statistically significant (p &lt; 0.001), (Fig. 2).

2

**Figure 3:**
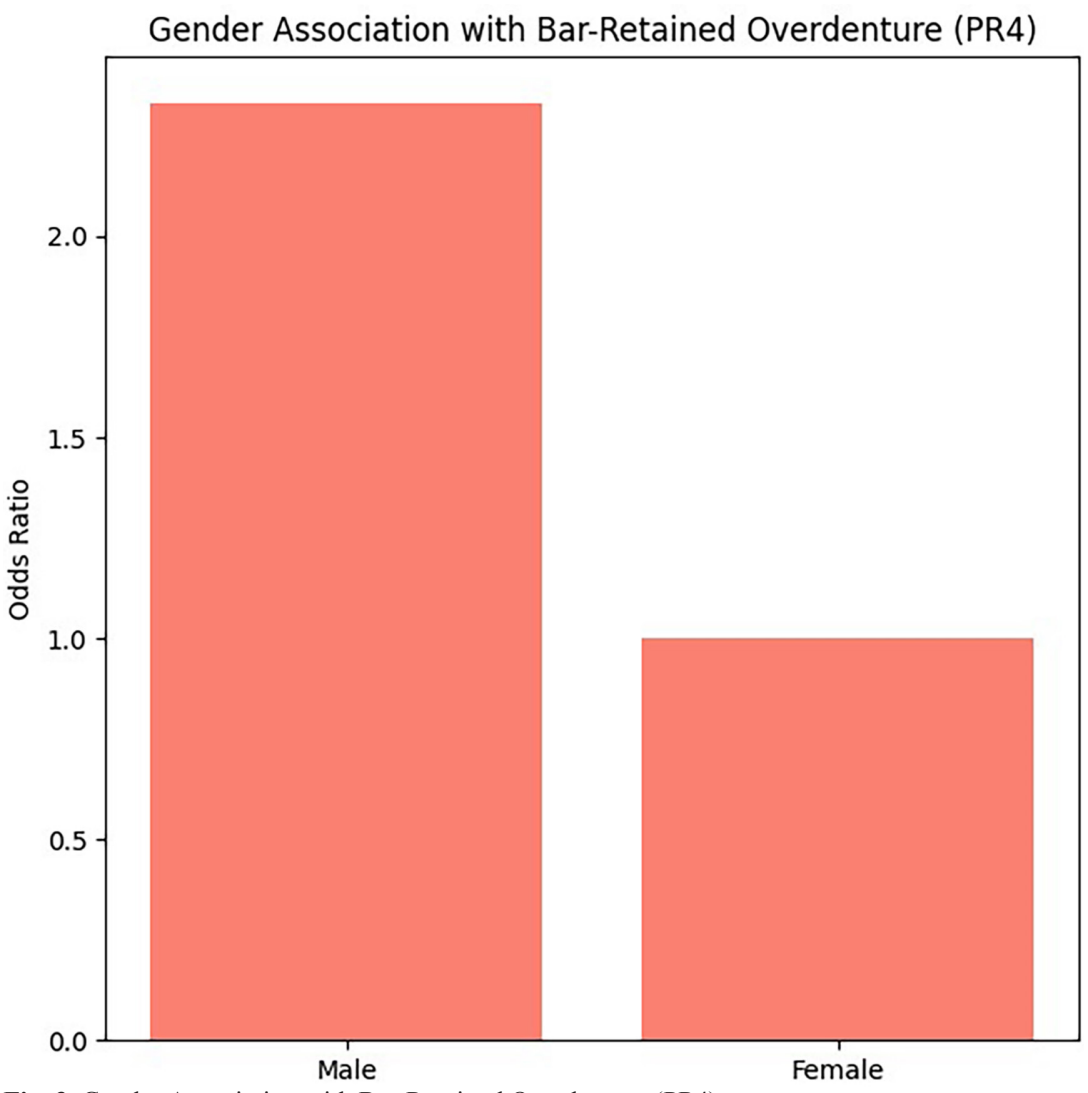
Gender Association with Bar-Retained Overdenture (PR4).

4. Sex and Prosthesis Type No statistically significant global association was observed between sex and prosthesis type in bivariate analysis. However, multivariate regression revealed that male patients had a significantly higher probability of being rehabilitated with bar-retained overdentures compared with single crowns, (Fig. 3).

3

## Discussion

This study provides an epidemiological analysis of implant-supported prosthetic rehabilitations performed in a Spanish university dental clinic over a five-year period. The large sample size allows for a robust evaluation of demographic patterns in prosthesis selection. The most consistent finding was the strong association between increasing age and the selection of more extensive prosthetic rehabilitations. Older patients were significantly more likely to receive fixed partial dentures or removable implant-supported prostheses, in line with previous epidemiological studies reporting higher rates of partial and complete edentulism with advancing age (12 - 14 , 20). The progressive annual increase in implant-supported treatments is consistent with global trends describing an expansion of implant dentistry and improved access to care, particularly within academic institutions (21 , 22). University clinics often provide implant rehabilitation to older populations seeking cost-effective treatment options. Sex-related differences were less pronounced, although male sex emerged as an independent predictor for bar-retained overdentures. Similar trends have been reported in studies suggesting higher rates of advanced tooth loss and risk behaviours among men, potentially influencing the selection of removable treatment modalities (15 , 16 , 23). The absence of detailed clinical and socioeconomic variables represents a limitation of this study. Nevertheless, the findings highlight the multifactorial nature of prosthesis selection and the relevance of demographic profiling in academic clinical settings.

## Conclusions

Implant-supported prosthetic treatments increased steadily between 2018 and 2022 in a Spanish university dental clinic. Single crowns and fixed partial dentures were the most frequently performed rehabilitations. Patient age was the strongest demographic predictor of prosthesis type, with older patients receiving more extensive fixed and removable rehabilitations. Male sex was independently associated with bar-retained overdentures.

## Data Availability

The data presented in this study are available from the corresponding author upon reasonable request.
